# DNA instability in replicating Huntington's disease lymphoblasts

**DOI:** 10.1186/1471-2350-10-11

**Published:** 2009-02-11

**Authors:** Milena Cannella, Vittorio Maglione, Tiziana Martino, Giuseppe Ragona, Luigi Frati, Guo-Min Li, Ferdinando Squitieri

**Affiliations:** 1 Neurogenetics Unit, IRCCS Neuromed, Pozzilli (IS), Italy; 2 Department of Experimental Medicine, University "Sapienza", Rome, Italy; 3 Department of Toxicology, University of Kentucky College of Medicine, Lexington (KY), USA

## Abstract

**Background:**

The expanded CAG repeat in the Huntington's disease (HD) gene may display tissue-specific variability (e.g. triplet mosaicism) in repeat length, the longest mutations involving mitotic (germ and glial cells) and postmitotic (neurons) cells. What contributes to the triplet mutability underlying the development of HD nevertheless remains unknown. We investigated whether, besides the increased DNA instability documented in postmitotic neurons, possible environmental and genetic mechanisms, related to cell replication, may concur to determine CAG repeat mutability. To test this hypothesis we used, as a model, cultured HD patients' lymphoblasts with various CAG repeat lengths.

**Results:**

Although most lymphoblastoid cell lines (88%) showed little or no repeat instability even after six or more months culture, in lymphoblasts with large expansion repeats beyond 60 CAG repeats the mutation size and triplet mosaicism always increased during replication, implying that the repeat mutability for highly expanded mutations may quantitatively depend on the triplet expansion size. None of the investigated genetic factors, potentially acting *in cis *to the mutation, significantly influence the repeat changes. Finally, in our experiments certain drugs controlled triplet expansion in two prone-to-expand HD cell lines carrying large CAG mutations.

**Conclusion:**

Our data support quantitative evidence that the inherited CAG length of expanded alleles has a major influence on somatic repeat variation. The longest triplet expansions show wide somatic variations and may offer a mechanistic model to study triplet drug-controlled instability and genetic factors influencing it.

## Background

The Huntington's disease (HD) mutation influences age at onset through its CAG repeat length, a genetic feature that is unstable during intergenerational parent-child transmission [1]. Transmitting males generally cause the highest expansions in successive generations. Expansion size progressively increases through a so-called multi-step mechanism [2,3], thus providing the molecular explanation for onset anticipation. Large CAG expansions above 60 repeats cause a severe phenotype leading to juvenile HD (JHD) [4]. The higher the expanded repeat length, the more instable is the triplet stretch in somatic and germline tissues [5-10]. In the brain of patients with another CAG expansion mutation disease, dentato-rubral pallido-luysian atrophy (DRPLA), dividing glial cells carry the largest CAG mutations [11,12], whereas, in HD, differentiating nonreplicating neurons carry the largest expansion mutations [13,14]. In other non-CAG triplet diseases with an excess of repeat expansions involving thousands of trinucleotides, somatic time-dependent variation in a CTG or GAA polymorphic stretch in the mutated alleles has also been documented in lymphoblasts, indicating lymphoblastoid cells as a valuable source for longitudinal analyses of triplet instability, mosaicism variability and genetic transmission [15,16].

Our purpose in this study was to investigate whether, besides the mechanisms influencing the CAG repeat mutability in HD terminally differentiated and nondividing neurons [13,14], cell division may contribute to DNA instability. To do so, we sought possible length- and time-dependent variability in the HD gene in serially passaged lymphoblastoid cell lines established from patients, after their passages in culture over time. We also studied the possible dependence of the somatic triplet variation and mosaicism (heterogeneity of the repeat length in the tissue) on CAG mutation length, on factors acting *in cis *or *in trans *to the mutation, and on drug-induced contraction of the mutation size.

## Methods

### Passaged cell lines and DNA study

Peripheral blood samples were collected after written informed consent. The subjects' consent was obtained according to the Declaration of Helsinki (Br Med J 1991; 302; 1194) after approval of the Bioethical Committee of Neuromed Institute. We obtained three blood samples from each subject; lymphocytes were isolated by differential centrifugation through Ficoll (Cederlane Laboratories) and transformed by Epstein Barr virus separately and in parallel from each sample as described [17] and according to the standard protocol [18]. A total 58 HD lymphoblastoid cell lines from subjects with a wide range of CAG expanded repeats, including low (39–41 CAG) and highly penetrant (60 CAG and more) mutations conventionally considered causing juvenile HD (Table 1), were serially passaged for at least 6 months (range: 6–12 months) to analyse longitudinal repeat variation during the passage time as previously described [17]. Groups of about 10 cell lines were cultured in parallel. All groups shared one identical clone obtained from one cell line as a marker of CAG size variability. This strategy was used to exclude potential length variability in the same cell cultures, and to highlight a potential influence on mutation size and mosaicism of yet unknown environmental factors during culture (for details see Additional file 1).

**Table 1:** Demographic characteristics of the patients and genetic characteristics of the lymphoblastoid cell lines in the patients with Huntington's disease.

Cell lines	No.	Gender Male/Female	Mean expanded CAG ± SD (range)	Mean Age at onset ± SD (range)	Over-time Triplet variation (ΔCAG)	Mean Mosaicism degree ± SD (No. peaks)	Expansions/contractions (triplet changes in %)
Lymphoblats with low mutation penetrance (**36–41 **CAG)	6	3/3	40.167 ± 0.983 (39–41)	57.250 ± 5.62 (50–63)	0.167 ± O.408 (0–1)	6.333 ± 1.966^a^ (5–10)	1/0 (1/6 or 17%)

Lymphoblats with usually expanded mutation penetrance (**42–59 **CAG)	43	22/21	45.628 ± 3.471 (42–54)	40.643 ± 10.094 (21–57)	0.628 ± 0.874 (0–3)	7.442 ± 2.797^b^ (3–14)	6/11 (17/43 or 39%)

Lymphoblasts with high mutation penetrance (**60–120 **CAG)	9	6/3	80 ± 21.042 (64–120)	12.750 ± 8.396 (3–25)	3.429 ± 1.512 (2 ≥ 5)	15.429 ± 11.238^c^ (7–40)	6/3 (9/9 or 100%)^d^

Total	58	31/27	50.397 ± 15.416 (39–120)	37.741 ± 14.819 (3–63)	0.929 ± 1.333 (0–5)	8.321 ± 5.250 (3–40)	13/14 (27/58 or 46%)

Greater CAG repeat expansions are associated with a larger number of CAG repeat changes (i.e. expansion/contraction events) in passaged lymphoblasts. DNA from passaged lymphoblasts with highest expansions of 60 CAG repeats or more causing juvenile Huntington's disease shows a greater maximum number of peaks (*p *> 0.001; c vs a and b) indicating the largest triplet mosaicism and a larger number of expansion events (d) than other cell lines.

For allele length analyses, genomic DNA was purified from blood lymphocytes, and lymphoblastoid cell lines containing about 1–3 × 10^6 ^cells, using standard procedures (see Additional file 1 and [17]). To elucidate potential factors acting either *in cis *or *in trans *with HD mutations, we also analysed CCG repeat size, deletion of the glutamic acid residue (ΔG) at nucleotide position 2642 of the HD gene, and the CAAΔCAG mutation in the 12 base pair region between CAG and CCG repeats, on both normal and mutated genes using previously described techniques [17]. All data concerning subjects' demographic features, cell-line mutation length and repeat number variation are included in Table 1.

### Analysis of somatic CAG repeat changes and mosaicism degree in passaged lymphoblasts

The GeneScan traces of the PCR amplification products from patients' passaged lymphoblastoid cell lines (see foregoing Methods) [17], appeared as a normal distribution composed of peaks differing in size by 3 bp and corresponding to a single CAG triplet change [8,19,20]. The size of the CAG repeat indicated by the highest peak corresponded to the most intense band obtained by hot PCR and was recorded as the size of the repeat in a given patient (see Additional file 2, Figure 1A). To determine the reproducibility of the CAG repeat size and its variation over time, we compared data yielded by hot and cold PCR techniques and sized samples on more than one gel. Somatic CAG repeat variation was defined by calculating the number of repeats either increased (e.g. expanded) or decreased (e.g. contracted) from the modal CAG repeat number of the expansion mutation in a given subject (see Additional file 2). Because some cell lines expanded or contracted during passages, the magnitude of the somatic CAG repeat variation in a given cell line was defined as the sum of both phenomena (expansions and contractions) occurring during the 6-month culture (see Additional file 3, Table 1). The repeat changes obtained in each cell line ranged between 0 in magnitude (no evidence of repeat changes over time, defined as ΔCAG = 0) and > 1 (ΔCAG >1), according to the number of expanded or contracted repeats or both, during 6-month culture, (see Additional files 1, 2, 3 and Table 1). The level of triplet mosaicism was expressed by the largest number of peaks calculated during the 6-month culture [8,19,20] (see Additional file 2). PCR products of normal alleles showed a single band/peak, always unchanged over-time, whereas expanded alleles consisted of multiple ladder bands/peaks, indicating somatic mosaicism. The size and range of expanded alleles varied among patients [8,19] and, sometimes, among the different cultured cell passages (see Additional file 2). To avoid artefacts potentially causing false bands or peaks due to the PCR procedure, we re-assessed the CAG size and its variation after serial dilution of DNA samples obtained from each cell line.

**Figure 1:**
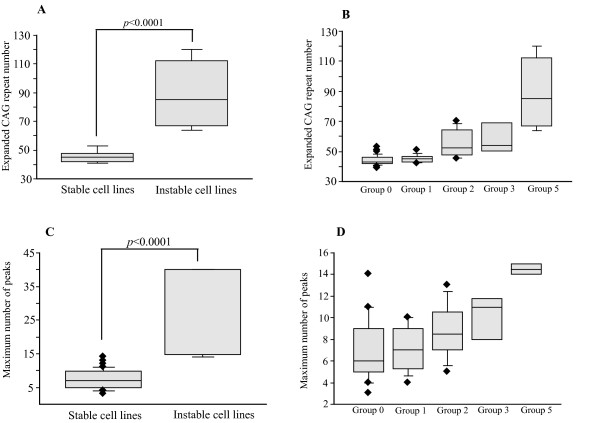
**Instable cell lines with (ΔCAG ≥ 5) and stable cell lines with no or small (ΔCAG ≤ 3) CAG repeat variation in dependence on expanded CAG repeat number and mosaicism**. A, Expansion CAG mutations are significantly larger in instable cell lines with ΔCAG ≥ 5 (n = 55; 89.4 ± 11.1 CAG) than in those with ΔCAG ≤ 3 (n = 55; 46.6 ± 0.10 CAG). B, Relationship between ΔCAG magnitude and expanded CAG repeat number. Groups 0, 1, 2, 3 and 5 represent cell lines with a ΔCAG value of 0, 1, 2, 3, and ≥ 5 repeats, respectively. The higher ΔCAG magnitude, the more significant is the statistical difference with Group 0-cell lines. Groups 2, 3 and 5 all showed a significant difference vs Group 0 (*p*-values = 0.0009, 0.0041 and < 0.0001, respectively). C, The mosaicism level, calculated by counting the maximum number of peaks, is significantly lower for stable cell lines with no or small repeat variation (n = 55, 7.4 ± 2.8 peaks and ΔCAG ≤ 3), than for those with large CAG changes (n = 5, 29.9 ± 13.7 and ΔCAG ≥ 5). D, The mosaicism level in dependence on ΔCAG: Samples with more than 80 CAG and about 40 peaks excluded as outliers to avoid a possible bias. Cell lines with large ΔCAG variations ≥ 5 CAG repeats showed an increased number of peaks and mosaicism vs each of the other groups with lower CAG variation (*p*-values = 0.0003, 0.0007, 0.0086 vs cell lines with ΔCAG = 0, 1 and 2 repeats, respectively). Diamonds in panels B, C and D represent outliers. Standard deviation is reported at the top of each bar.

### Cell-line drug treatment

In an attempt to reduce the mutation size and stability, we tested drugs influencing some of the suggested mechanisms potentially related to triplet instability [21]. For drug treatment experiments, two "stable" and two particularly "unstable" and prone-to-expand cell lines (of 74 and 85 CAG repeats) were selected. We treated cell lines with the following drugs: ethylmethanesulphonate (EMS), a GC/AT modifier thought to prevent CAG expansion in lymphoblasts derived from patients with myotonic dystrophy type 1 [22]; ethidium bromide (EB), a DNA intercalating drug reducing the rate of repeat expansion by inhibiting enzymes that bind to DNA [23]; and mitomycin C (Mit-C), an interstrand crosslinker [22]. Each progenitor culture was split into multiple aliquots: four flasks for drug treatment (one each for EMS, EB and Mit-C) and one flask for control. Each treatment was performed in duplicate. All cultures were serially passaged as previously described and maintained in parallel throughout the experiments (for further cell line culture and drug treatment methodology see Additional file 1).

### Statistical analysis

For statistical analysis we used nonparametric tests: Mann-Whitney U test, to compare differences between cell lines with different ΔCAG (small triplet changes with ΔCAG ≤ 3 vs large triplet changes ≥ 5), and Kruskal-Wallis test to compare differences across more than two groups. A simple regression model was used to test the linear dependence of the maximum number of peaks (CAG mutation mosaicism) on expanded CAG repeats. Data are presented as means ± SE. Statistical analysis was performed with Stat View V (tests considered significant at *p *≤ 0.05).

## Results

### Expanded CAG repeat size-dependent changes

Triplet variation varied over culture time in a CAG length-dependent manner (Table 1). Most passaged cell lines showed no or minimal (1 to 3 repeats) over-time triplet variation (51/58 cell lines or 88%; Table 1), including small contraction or expansion events. The repeat changes obtained in each cell line ranged between 0 in magnitude (ΔCAG = 0, n = 31) and > 1 [ΔCAG > 1, n = 27; Group 1 (n = 11), 2 (n = 8), 3 (n = 3), or ≥ 5 (n = 5)], according to the number of expanded or contracted repeats or both, during 6-month culture (see Additional files 1, 2, 3 and Table 1). Cultured cell lines showing over-time triplet variation (and ΔCAG greater than 5) had, on average, a larger CAG repeat number than cell lines with no or little somatic variation (*p *< 0.0001, Figure 1A). The magnitude of ΔCAG significantly correlated with the size of the expanded CAG repeat: the more expanded the repeat number was, the higher were the ΔCAG values (*p *values ranged from 0.0041 to < 0.0001, Figure 1B). All cell lines, regardless of their ΔCAG values, exhibited some degree of triplet mosaicism (i.e., repeats varied in size). The degree of mosaicism invariably increased significantly in cell lines carrying large expansions and ΔCAG ≥ 5 (*p *< 0.0001, Figure 1C). The maximum number of peaks (e.g. the level of triplet mosaicism indicating a mutation rate degree in a certain tissue) significantly correlated with ΔCAG magnitude (Figure 1D).

No significant association of investigated *in cis *and *in trans *factors influenced the repeat variation in our cohort of cell lines (see Additional file 1). Nor did we ever observe the CAAΔCAG mutation in the 12 base-pair region between CAG and CCG repeats, on either normal or mutated genes, regardless of ΔCAG values.

### Drug-induced CAG contraction

**Figure 2:**
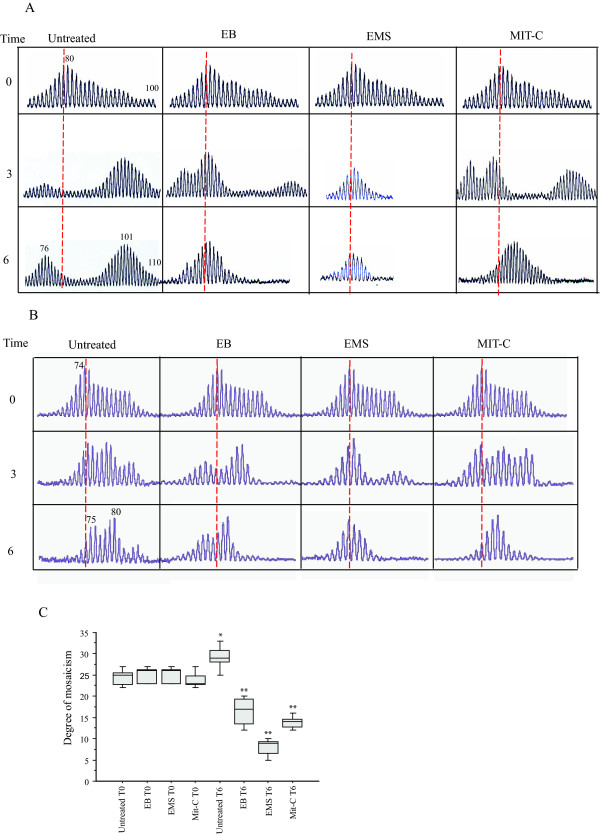
**Drug treatment in lymphoblastoid cells with a highly expanded mutation**. A and B, Two untreated highly expanded and prone-to-expand cell lines (panel A: cell line with 80 CAG repeats; panel B: cell line with 74 CAG repeats) show an increased CAG repeat variation. The 6^th ^time yielded two peaks with a large level of mosaicism in both cell lines, in one (A) extended beyond 100 CAG repeats. Treatment with ethidium bromide (EB), ethylmethanesulphonate (EMS) and mitomycin C (Mit-C) progressively restricted the mosaicism and the expanded triplet variation in both cell lines. C, Mosaicism variation in treated and untreated cell lines. Untreated cell lines significantly increased their mosaicism degree observed at time 6 vs time 0 (*p = 0.019, see online/supplementary Table 3). Treated cell lines at time 6 significantly decreased their mosaicism degree vs time 0 (**p < 0.0001, see Additional file 1). Degree of mosaicism: median number of peaks observed at time 0 and 6 from two untreated and treated cell lines carrying large CAG repeat expansions. Statistical analysis performed by ANOVA. EB = ethidium bromide, EMS = ethylmethanesulphonate; Mit-C = mitomycin C.

In experiments designed to explore whether drug treatment suppressed somatic repeat expansion [21,24], using lymphoblastoid cell lines derived from HD patients as a model, we treated two expansion-prone cell lines with EB, EMS and Mit-C, chemicals or drugs thought capable of reducing triplet expansion [22,25] (Figure 2). All the chemicals we used, EB, EMS, and Mit-C, considerably reduced CAG repeat numbers, especially in cells treated for 6 months. The drug-induced contraction apparently eliminated the bi-modal intra-allele CAG distribution in both cell lines, suggesting that triplet expansion is preventable. The same drugs failed to influence the size of the unexpanded alleles in both cell lines (see Additional files 4 and 5).

## Discussion

The relatively small magnitude of triplet changes and length gains we observed in most cell lines (88% of total) confirms that cell-division related events contribute minimally to HD instability and is in agreement with the recent findings concerning CAG repeat changes mainly occurring in terminally differentiating nondividing neurons [13,14,26]. The only exception in our model were the very large expansion mutations showing increased over-time gains of CAG variations in dividing cells (see Additional file 2, Figures 1 and 2, Table 1). Lymphoblasts with highly expanded mutations are particularly prone to oxidative stress [27,28] and decreased mitochondrial ATP production [29]. More recent research has proposed a model taking into account a relationship between oxidative lesions accumulating in brain and progressive somatic gains in expanded repeats resulting from errors in repairing [26]. Hence besides mechanisms determining DNA instability as reported in differentiating neurones [13,14,26], CAG mutability might have arisen also from an age related oxidative-stress in highly expanded lymphoblasts ample evidence shows that JHD patients show extended brain damage associated with a particularly severe phenotype [4] and large mutations cause JHD, we conjecture that the cell division occurring in glial cells with large mutations may contribute to an excess of expanded repeat gains, thus contributing to more widespread brain disease, as happens in DRPLA [11,12]. This observation is in line with the hypothesis that in certain cases of HD, mutation length gains may continue to accumulate during life, as the disease progresses [30].

Consistent with a previous report [17], we found no influence on instability of the analysed factors potentially acting *in-cis *to the mutation (see Additional file 1), including the CAAΔCAG mutation in the 12 bp region between the CAG and CCG repeats, described by Goldberg et al. [7]. This apparent discrepancy may depend on the fact that Goldberg et al. studied a population of variable ethnic origin whereas ours was an ethnically homogeneous population [17]. Because all our cell lines showed the CAA codon on both alleles in the 12 bp region between the CAG and CCG repeats, regardless of the CAG repeat variation, we therefore rule out even the potential effect of such a factor potentially acting in *trans *with the CAG repeat instability in our population. The role of the normal allele size to test the hypothesis of whether the CAG repeat polymorphism in the non-HD range is a potential physiological modifier of mutation instability [29,31-33] remains to be investigated.

To reduce *in vitro *the mutation length of particularly large and unstable expansions and to approach a potential therapeutic strategy, we treated two cell lines with drugs thought capable of influencing mechanisms of instability [21,22,25]. When we used drugs interfering with diverse mechanisms to treat two cell lines carrying large mutations and particularly prone-to-expand, the expanded (but not the normal allele) repeat number contracted, and the bi-modal intra-allele CAG distribution progressively disappeared (Figure 2). Hence, we presume that many different mechanisms act in concert to influence CAG repeat instability. Given that somatic repeat expansion affects the HD phenotype, this finding provides useful information insofar as contracting the longest repeat number is among the putative pharmacological strategies for use in patients with HD, particularly when high expansion mutations cause early age at onset [22,25]. Intriguing questions left open for future research include the mechanisms underlying triplet repeat expansion (e. g. replication, recombination and repair processes), and their drug-induced contraction.

The main limitation of our model is probably cell proliferation induced by an infecting virus. Indeed, as recently demonstrated in HD patients, mutation length gains of CAG repeats may even occur in somatic cells or neurons well after these cells are terminally differentiated and mitotic replication has ceased. The fact that our proliferating cell model mostly disclosed small contraction/expansion changes in few triplets nevertheless theoretically offers further strength and support to evidence that repeat length gains may occur independently of replication, as demonstrated in the striatal neuronal population [13,14,26]. An additional limitation of our work may depend on the limited (two) repetitions of the experiments. This was due to the relatively large number of cell lines cultured for long times (at least six months) with a relevant number of PCR assays required and performed each week (see Methods and Additional file 1). Therefore, the inter-experimental reproducibility is unknown as statistical significance could not be determined from the limited (two) repetitions of the experiments. Despite the potential limitations of this and other studies [15,16,27-29,34], i.e. the biological influence of the infecting virus on immortalised cells and the inter-experimental reproducibility, our study may offer an *in vitro *approach to human cells in the attempt to provide a new experimental model system for mechanistic studies of triplet expansion (mitotic vs repair synthesis or other mechanisms so far reported) and clinical treatments for HD.

## Conclusion

Our study offers further relevance to the hypothesis that the repeat mutability depends quantitatively on CAG triplet expansion size. The longest triplet expansions show wide somatic variations and may offer a mechanistic model to study triplet drug-controlled instability. Replication does not affect somatic variability in most of the cell lines whose CAG expansion size ranges in the usual mutation penetrance confirming that cell-division related events contribute minimally to HD instability, in agreement with the findings concerning CAG repeat changes mainly occurring in terminally differentiating nondividing neurons. Instead, large repeat expansions causing JHD tend to expand during replication supporting the hypothesis that mechanisms affecting CAG mosaicism and mutability may differ in dependence on the mutation length, in certain tissues, and as the disease progresses. In our experiments certain drugs controlled triplet expansion in prone-to-expand HD cell lines carrying large CAG mutations. Finally, we found no influence on instability of the analysed factors potentially acting either *in cis *or *in trans *with the mutation, including the CAAΔCAG mutation in the 12 bp region between the CAG and CCG repeats.

## Competing interests

The authors declare that they have no competing interests.

## Authors' contributions

GR and FS Study concept and design. FS Drafting of the manuscript. MC, TM Acquisition of molecular genetic data. VM and GML Drug-treatment of cell lines. VM Lymphoblastoid cell line bank and virus infection. MC Art work and manuscript editing. MC, VM, GML, GR, FS Analysis and interpretation of data and statistical analysis. MC, VM, TM, GML, GR, LF, FS Critical revision of the manuscript for important intellectual content. LF, MC, TM Administrative, technical and material support. LF and FS Study supervision.

## Pre-publication history

The pre-publication history for this paper can be accessed here:



## Supplementary Material

Additional file 1 **Methods, Individual cell lines from each subject, Polymorphisms analysis and statistical analysis.** Click here for file

Additional file 2 **GeneScan traces of CAG repeats in HD lymphoblastoid cell line.** Click here for file

Additional file 3 **GeneScan traces of CAG repeats in HD lymphoblastoid cell line.** Click here for file

Additional file 4 **Drug treatment in lymphoblastoid cells, effect on unexpanded alleles** Click here for file

Additional file 5 **Drug treatment in lymphoblastoid cells effect on unexpanded alleles** Click here for file
