# Supplementary material for: DNA instability in replicating Huntington's disease lymphoblasts
Source: BMC Med Genet. 2009 Feb 11;10:11. doi: 10.1186/1471-2350-10-11 (PMC2645380; doi:10.1186/1471-2350-10-11)
Supplement: Additional file 1 — Methods, Individual cell lines from each subject, Polymorphisms analysis and statistical analysis. [file 1471-2350-10-11-S1.doc]

**Additional file 1 material and methods**

**Lymphoblast cultures.** Cells were maintained in suspension in 25-cm^2^ flasks in RPMI 1640 (Invitrogen), supplemented with 10% fetal bovine serum (FBS), penicillin/streptomycin (Invitrogen), and L-glutamine (Invitrogen). At each passage (twice per week), 3 x 10^6^ cells were transferred to 8 ml fresh medium after thorough resuspension. After 8 to 10 similar passages (15-20 doublings), a portion of the cells was used for isolation of genomic DNA. In total, these cells underwent about 60 passages during 6-month culture.

**Analysis of repeat sizes by polymerase chain reaction.** The CAG repeat number in the *HTT* gene and its variation were analyzed using a GC–rich PCR amplification Kit (Invitrogen) and primers that include (total CAG) or exclude (pure CAG) the polymorphic CCG repeat stretch, as described elsewhere [4]. (PCR products were analysed by polyacrylamide gel electrophoresis to determine radiolabelled PCR products or with an ABI Prism 3100 Genetic Analyzer to determine dye-tagged products, followed by GeneScan 3.1 software.

**Cell line cultures for drug treatments.** Drug treatments were started after cell lines had been cultured for one month. All lymphoblastoid cell lines were treated with drugs in growth media at
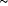
1 x 10^6^ cells/ml for various times. Cells were treated with EMS (700 µg/ml) for 6 h or with Mit-C (0.2 µg/ml) for 14 h [22]**,** washed once with 1X PBS, and resuspended in fresh medium, followed by continuous culture for 5 months. EB-treated cells were cultured for 5 months in culture media containing 250 nM Et-Br [13]. All cells were serially passaged about ~ 10 time a month as described. At selected passages [8-10], an aliquot of cell cultures was used for DNA isolation. In EMS and Mit-C experiments, a second dose of the drug was added to the culture medium in the same manner after about 20 serial passages in culture and cells were further cultured for three months.

**Additional Table 1. Individual cell lines from each subject**

| **Low penetrance cell lines**  **36-41 CAG** |  |  |  |  |
| --- | --- | --- | --- | --- |
| **Subject's initials** | **Nb** | **Expanded CAG** | **Repeat changes** | **Events** |
| SG | 1 | 36 | Group for 0 | no |
| RL | 2 | 39 | Group for 0 | no |
| DPN | 3 | 39 | Group for 0 | no |
| AL | 4 | 40 | Group for 0 | no |
| SG | 5 | 41 | Group for 0 | no |
| RM | 6 | 41 | Group for 1 | exp |
|  |  |  |  |  |
| **Usual penetrance cell lines**  **42-59 CAG** |  |  |  |  |
| **Subject's initials** | **Nb** | **Expanded CAG** | **Repeat changes** | **Events** |
| SC | 1 | 41 | Group for 0 | no |
| AS | 2 | 42 | Group for 0 | no |
| DG | 3 | 42 | Group for 0 | no |
| CA | 4 | 42 | Group for 0 | no |
| CM | 5 | 42 | Group for 0 | no |
| MMG | 6 | 42 | Group for 0 | no |
| BP | 7 | 42 | Group for 0 | no |
| BL | 8 | 42 | Group for 0 | no |
| EMV | 9 | 42 | Group for 0 | no |
| DLL | 10 | 42 | Group for 0 | no |
| DMI | 11 | 43 | Group for 0 | no |
| CR | 12 | 43 | Group for 0 | no |
| LG | 13 | 44 | Group for 0 | no |
| SE | 14 | 44 | Group for 0 | no |
| BR | 15 | 44 | Group for 0 | no |
| DML | 16 | 44 | Group for 0 | no |
| ZA | 17 | 44 | Group for 0 | no |
| ZD | 18 | 44 | Group for 0 | no |
| CF | 19 | 45 | Group for 0 | no |
| LB | 20 | 46 | Group for 0 | no |
| SS | 21 | 46 | Group for 0 | no |
| RE | 22 | 46 | Group for 0 | no |
| MV | 23 | 47 | Group for 0 | no |
| TE | 24 | 48 | Group for 0 | no |
| VI | 25 | 51 | Group for 0 | no |
| MA | 26 | 53 | Group for 0 | no |
| MA | 27 | 42 | Group for 1 | contr |
| KR | 28 | 43 | Group for 1 | exp |
| CG | 29 | 43 | Group for 1 | contr |
| TR | 30 | 45 | Group for 2 | contr |
| CC | 31 | 45 | Group for 1 | exp |
| AF | 32 | 45 | Group for 1 | contr |
| LT | 33 | 45 | Group for 1 | contr |
| PL | 34 | 45 | Group for 1 | contr |
| GT | 35 | 47 | Group for 1 | contr |
| MP | 36 | 47 | Group for 1 | contr |
| CA | 37 | 51 | Group for 1 | exp |
| SE | 38 | 49 | Group for 3 | exp |
| VA | 39 | 47 | Group for 2 | contr |
| SG | 40 | 48 | Group for 2 | exp |
| RL | 41 | 52 | Group for 2 | contr |
| PG | 42 | 53 | Group for 2 | contr |
| VF | 43 | 54 | Group for 3 | exp |
|  |  |  |  |  |
| **High penetrance cell lines**  **60-120 CAG** |  |  |  |  |
| **Subject's initials** | **Nb** | **Expanded CAG** | **Repeat changes** | **Events** |
| FG | 1 | 64 | Group for 2 | contr |
| VE | 2 | 70 | Group for 2 | exp |
| MG | 3 | 72 | Group for 3 | exp |
| CA | 4 | 74 | Group for 3 | exp |
| CG | 5 | 64 | Group for 5 | contr |
| LP | 6 | 68 | Group for 5 | contr |
| LM | 7 | 85 | Group for 5 | exp |
| OR | 8 | 110 | Group for 5 | exp |
| PP | 9 | 120 | Group for 5 | exp |

Data from each subject’s cell line is reported according to the mutation penetrance, in line with the data reported in Table 1. contr=contraction, exp=expansion, no=no expansions or contractions.

**Additional Table 2.** Polymorphisms analysis in Huntington’s disease gene

| **Alleles** | **No.**  **%** | **CCG repeat number**  **7 8 9 10** | **ΔG**    **A B** |
| --- | --- | --- | --- |
| With no somatic expanded CAG variation (∆CAG = 0) | 35  30% | 32 3  91% - - 9% |  |
| With somatic expanded CAG variation (∆CAG > 0) | 28  24% | 22 6  79% - - 21% | 24 4  86% 14% |
| With unexpanded CAG repeats and ∆CAG = 0 on the mutated gene | 26  23% | 18 3 5  69% 12% - 19% | 26 0  100% 0% |
| With unexpanded CAG repeats and ∆CAG > 0 on the mutated gene | 27  23% | 19 8  70% - - 30% | 26 0  100% 0% |
| Total | 116  100% | 91 3 - 22  78% 3% 19% | 109 6  95% 5% |

Genetic factors potentially acting *in* *cis* or *in* *trans* with the CAG mutation failed to show any significant influence on expanded repeat variation.

**Additional Table 3. Statistical difference (ANOVA) in the median number of peaks from two cell lines (in five observations) observed at time 0 and time 6.**

**Legends to Additional files 2, 3, 4 and 5**

**Additional files 2 and 3**. GeneScan traces of CAG repeats in Huntington’s disease lymphoblasts. A, GeneScan traces of serially passaged cell lines showing no CAG repeat variation (Panels A, D, G, J) and an increase in 1 CAG repeat over time Panels B, E, H, K). Panels C, F, I: GeneScan traces showing CAG repeat mosaicism modification of a highly expanded cell line with 80 CAG repeats, causing juvenile Huntington’s disease. B, GeneScan traces of a serially passaged cell line showing contraction of 1 CAG repeat (Time III) and further expansion of 1 repeat (Time IV), thereby yielding a ∆CAG magnitude of 2. Panel B shows only the enlarged expanded allele.

**Additional files 4 and 5.** Drug treatment in lymphoblastoid cells: effect on unexpanded alleles. A and B, Unexpanded alleles of the highly expanded cell lines (enlarged expanded alleles reported in Figure 2) failed to show any modification after drug treatments with ethidium bromide (EB), ethylmethanesulphonate (EMS) and mitomycin C (Mit-C). A, Unexpanded allele (18 CAG) associated with CAG expansion of 80 repeats (enlarged in Figure 2A). B, Unexpanded allele (21 CAG) associated with CAG expansion of 74 repeats (enlarged in Figure 2B).
